# Ground kinematically aligned total knee arthroplasty: new personalized technique which enables a stable knee with deep flexion

**DOI:** 10.1007/s00264-023-05799-6

**Published:** 2023-04-22

**Authors:** Tomoyuki Matsumoto, Naoki Nakano, Kazunari Ishida, Yuichi Kuroda, Shinya Hayashi, Hirotsugu Muratsu, Ryosuke Kuroda

**Affiliations:** 1 Department of Orthopedic Surgery, Kobe University Graduate School of Medicine, 7-5-1, Kusunoki-Cho, Kobe, Chuo-Ku 650-0017 Japan; 2 Department of Orthopedic Surgery, Kobe Kaisei Hospital, Kobe, Japan; 3 Department of Orthopedic Surgery, Hyogo Prefectural Harima-Himeji General Medical Center, Himeji, Japan

**Keywords:** Kinematically alignment, Total knee arthroplasty, Calcaneus, Ground mechanical axis

## Abstract

**Purpose:**

This study aimed to evaluate kinematically aligned total knee arthroplasty (KA-TKA) targeting the neutral ground mechanical axis (MA) (hip-to-calcaneus axis), the line from the hip centre to the bottom of the calcaneus, (ground KA-TKA) in terms of its comparison with tibia-restricted modified KA-TKA (modified KA-TKA).

**Methods:**

This retrospective cohort study included 106 consecutive patients who underwent unilateral KA-TKA for varus osteoarthritis (OA) (60 modified KA-TKAs and 46 ground KA-TKAs). After 1:1 propensity score matching, 60 patients (30 pairs) were matched between the groups with comparable demographic data. The hip-knee-ankle (HKA) angle, coronal femoral component alignment (FCA), and coronal tibial component alignment (TCA) were compared between groups. Intraoperative soft tissue balance, including the joint component gap and varus/valgus balance, was also compared between the groups. One year postoperatively, the clinical outcomes, including the range of motion and 2011 Knee Society Score, were compared between groups.

**Results:**

The HKA angle and FCA/TCA were not significantly different between groups. Whereas the varus/valgus balance showed no significant differences between groups, smaller joint component gaps were found throughout the range of motion in the ground KA-TKA group than in the modified KA-TKA group. Despite no difference in clinical scores between groups, a significantly deeper postoperative flexion angle was achieved in the ground KA-TKA group than in the modified KA-TKA group (*p* < 0.05).

**Conclusion:**

Targeting neutral ground MA in KA-TKA for patients with varus OA has the potential to provide a better flexion angle with stable intraoperative soft tissue balance.

## Introduction

The alignment philosophy in total knee arthroplasty (TKA) has recently shifted from mechanical alignment as the gold standard to personalized alignment [1]. Among them, anatomical and restricted kinematically aligned (KA) TKA [2, 3] have gained popularity for reproducing physiological joint lines and kinematics with minimal soft tissue release. However, recent meta-analyses have shown that the advantage in KA TKA is still controversial compared with mechanically aligned TKA; one showed better early clinical outcomes and the other did not [4, 5].

The hip-to-calcaneus axis, known as the ground mechanical axis (MA) [6, 7], passes through the center of the knee joint in the native knee [8]; it has attracted attention as an alternative alignment assessment to the hip-to-talus axis, the gold standard MA [9–11]. Matsumoto et al. reported a tibia-restricted modified KA-TKA procedure [12], where the femoral component was placed on the cylindrical axis using the calipered technique, and the tibial component constantly placed at 3° varus. On average, this procedure resulted in joint lines parallel to the ground and a similar alignment to that in young healthy individuals [8], with the ground MA unexpectedly passing through the center of the knee joint. Furthermore, Kamenaga et al. performed a gait analysis and reported that the hindlimb plantar pressure distribution after tibia-restricted modified KA-TKA is similar to that in normal individuals [13]; that is not the case in mechanically aligned TKA. Hence, for individualized reproduction of native limb alignment and knee kinematics [14], ground MA may be an alternative target, particularly for KA-TKA.

Recently, a new concept of KA-TKA targeting the neutral ground KA for patients with varus osteoarthritis (OA) (ground KA-TKA) was introduced with a feasible technique and fewer alignment outliers, and was reported to potentially provide a physiological alignment, more similar to that of the native knee than modified KA-TKA [15]. However, details, including intraoperative soft tissue balance and postoperative outcomes, remains unclear. Therefore, this study aimed to compare radiographic, intraoperative soft tissue balance, and early-term clinical outcomes of ground KA-TKA and tibia-restricted modified KA-TKA in patients with varus OA. The hypothesis of the study was that the ground KA-TKA would reproduce more stable intraoperative soft tissue balance and better clinical outcomes compared with the modified KA-TKA, because the ground KA-TKA technique individually fits the anatomical differences of each knee.

## Materials and methods

### Radiographic simulation for the ground KA-TKA technique

**Fig. 1:**
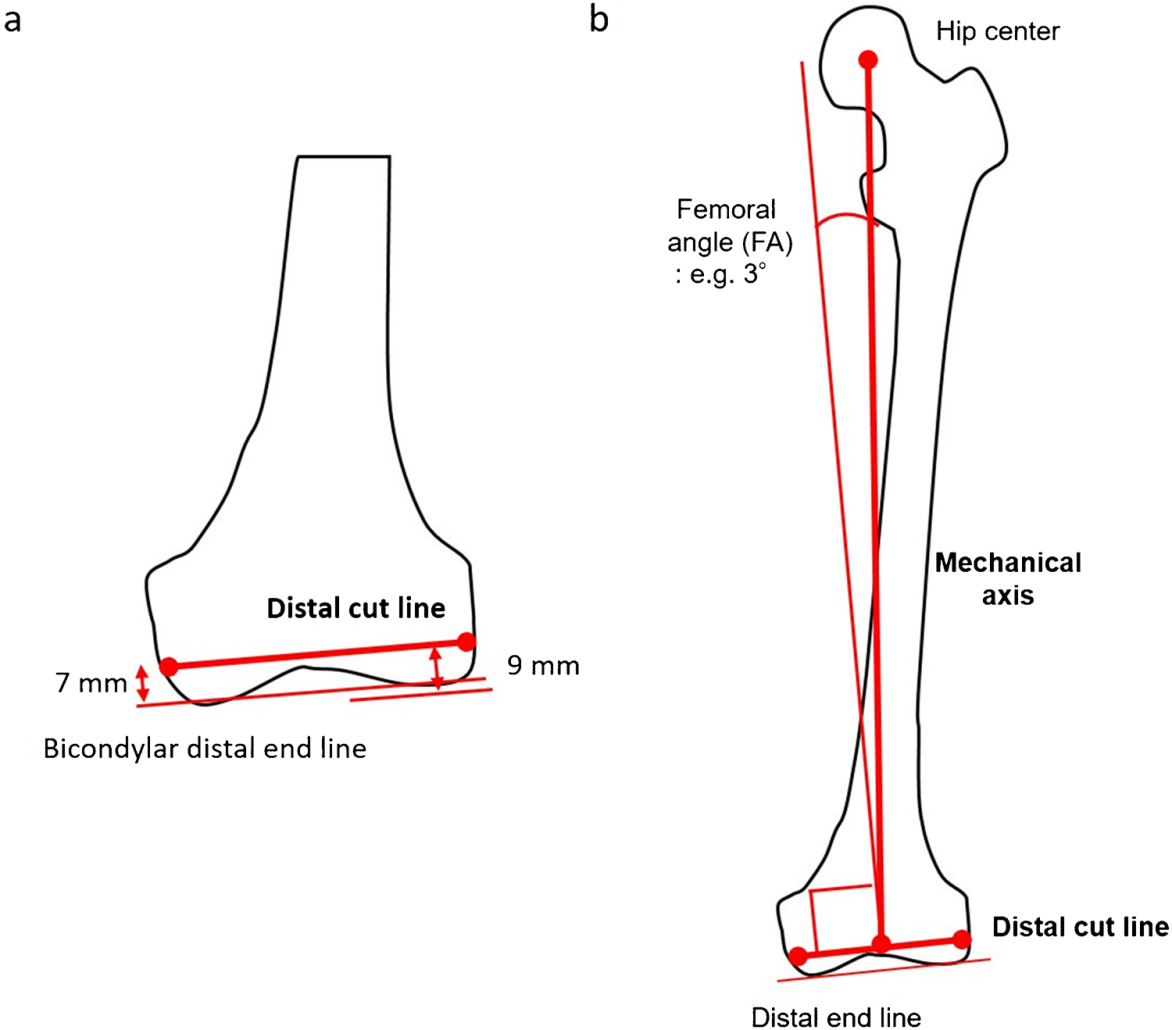
Distal femoral bone cut simulation. **a** The femoral bone cut line was simulated to be 7 mm proximal to and parallel to the bicondylar distal end line. Considering a 2-mm cartilage thickness, the medial side with cartilage wear and the lateral side with invisible cartilage were 7 mm each (equal to a 9-mm distal thickness of the femoral component). **b** The mechanical axis from the hip center to the center of the distal cut line generally results in valgus in relation to the perpendicular line of the distal cut line. In this case, the femoral angle is 3° valgus

To simulate ground KA-TKA, the femoral distal and tibial proximal cut lines were first simulated using full-length standing coronal radiography that included the calcaneus. The femoral distal cut was 9 mm thick on the lateral side and 7 mm thick on the medial side, as per the calipered technique. Considering cartilage thickness, the distal cut line was simulated to be 7 mm proximal to both the medial and lateral sides of the bicondylar distal end line (Fig. 1a). The MA of the femur was from the hip center to the center of the distal cut line. The femoral angle (FA) was defined as the angle between the MA and the line perpendicular to the distal cut line and was measured: this is commonly the valgus angle in varus-type OA (Fig. 1b).

**Fig. 2:**
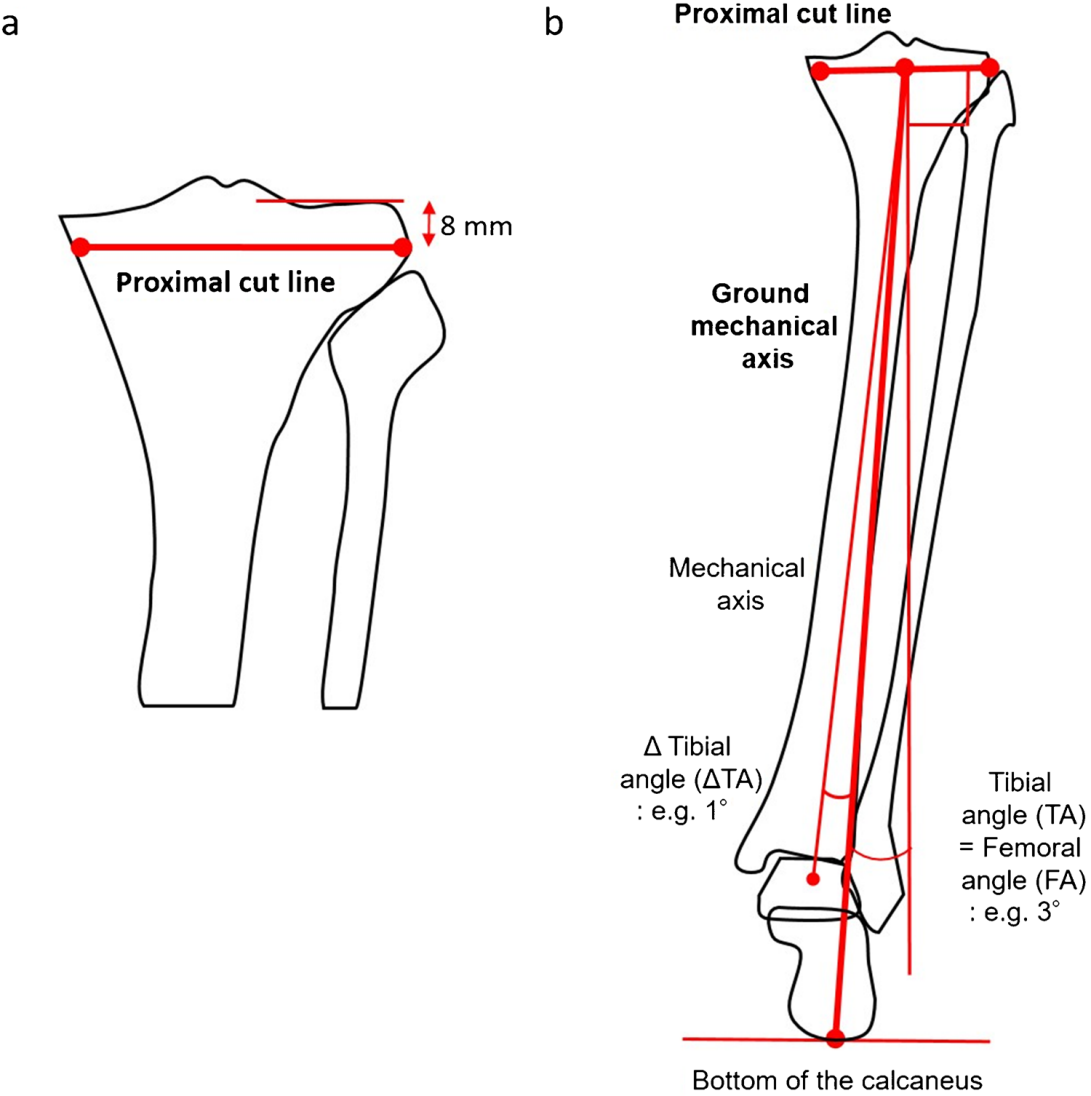
Proximal tibial bone cut simulation. **a** The proximal cut line was 8 mm distal to the lateral joint line of the tibia. Considering a 2-mm cartilage thickness, the lateral side with invisible cartilage was 8 mm (equal to a 10-mm thickness of the tibial component). **b** The proximal cut line was simulated to neutralize the femoral angle in relation to the ground mechanical axis from the center of the proximal cut line to the bottom of the calcaneus. The navigation system refers to the mechanical axis and not ground mechanical axis. The mechanical axis is typically medial to the ground mechanical axis. If the ankle is located 1° medial to the calcaneus (ΔTA = 1° varus) and the FA is 3° valgus (TA = 3° varus), the navigated tibial cut angle should be 4° varus

For the simulation of the tibial side, considering the 2-mm thickness of the cartilage, the proximal cut line was 8 mm distal to the lateral joint line of the tibia (Fig. 2a). The proximal cut line was set to neutralize the FA in relation to the ground MA from the centre of the proximal cut line to the bottom of the calcaneus. If the resulting FA is 3° valgus to the MA of the femur, a 3° varus tibial cut in relation to the ground MA of the tibia as the tibial angle (TA) should be performed with the assistance of the navigation system. The navigation system refers to the ankle centre rather than the bottom of the calcaneus. Therefore, ΔTA was defined as the angle between the MA and the ground MA of the tibia and was measured; this parameter should also be measured preoperatively. Generally, the calcaneus is located lateral to the ankle centre. If the FA was 3° valgus (TA = 3° varus) and the bottom of the calcaneus was 1° lateral to the ankle center (ΔTA = 1° varus), the navigated tibial cut angle (nTA) was 4° varus (Fig. 2b). These parameters were calculated as follows: “nTA (varus) = TA (varus)(FA (valgus)) + ΔTA (varus).”

### Ground KA-TKA compared with modified KA-TKA

The hospital’s ethics committee approved the study protocol (No. 290038), and the patients provided informed consent for participation in the study. The inclusion criteria were as follows: substantial pain and loss of function due to severe OA of the knee (Kellgren–Lawrence grade 3–4), with a functional posterior cruciate ligament (PCL) based on the intercondylar osteophytes assessed by preoperative epicondylar view radiograph and computed tomography. To make a fair assessment and minimize the influence of clinical variables, the exclusion criteria included the following: knees with valgus deformity, severe varus deformity > 20°, flexion contracture > 20°, revision TKAs, active knee joint infections, and the need for bilateral TKA. To avoid compensatory hindfoot alignment change postoperatively [16, 17], patients with prior ankle or foot surgery, foot or ankle deformity (such as flat foot, hallux valgus, and ankle OA), history of ankle fracture, and those unable to stand stably on one leg for > 10 s without support were also excluded. Between January 2019 and December 2021, 106 consecutive patients who met the abovementioned criteria were prospectively enrolled in this study and underwent cruciate-retaining TKA (Persona®. Zimmer Inc., Warsaw, IN, USA) using a portable navigation system (iASSIST® Zimmer-Biomet Japan Inc., Tokyo, Japan). This cohort included 60 consecutive tibia-restricted modified KA-TKAs performed from January 2019 to December 2020, and 46 consecutive ground KA-TKAs performed from January to December 2021. All operative procedures were performed by a senior surgeon (T.M.) with > 15 years of experience in performing TKAs. To adjust for baseline patient characteristics between groups, 1:1 propensity score matching was used based on logistic regression models using age, sex, body mass index, preoperative deformities, preoperative range of motion (ROM), and subscales of the patient-derived 2011 Knee Society Score (KSS) [18] (objective indicators, patient satisfaction, and functional activity scores). Thirty matched pairs were finally enrolled in this study (Table 1).

**Table 1:** Patient demographic data after propensity score matching

	Ground KA-TKA	Modified KA-TKA	*p*-value
Number	30	30	
Age (years)*	73.5 ± 8.4	72.0 ± 7.8	0.487
Sex (% female)	89.7.0% (26/30)	82.8% (24/30)	0.706
Body mass index*	26.5 ± 4.9	27.0 ± 2.8	0.658
Deformity (varus) (degree)*#	11.2 ± 2.9	12.2 ± 4.9	0.314
Range of motion			
Extension angle (degree)	− 12.8 ± 5.7	− 11.7 ± 4.3	0.456
Flexion angle (degree)	120.3 ± 12.2	120.2 ± 12.3	0.957
2011 KSS			
Objective indicator*	65.2 ± 7.9	65.7 ± 11.7	0.854
Patient satisfaction*	13.4 ± 5.6	12.7 ± 7.3	0.670
Functional activity score*	47.1 ± 12.0	46.0 ± 20.1	0.794

^*^Data are presented as mean ± standard deviation

^#^Positive values indicate varus alignment

*KA-TKA* kinematically aligned total knee arthroplasty, *KSS* Knee Society Score

### Operative procedures

After inflating the air tourniquet to 250 mm Hg, medial parapatellar arthrotomy was performed. All surgeries were performed using the extension-gap-first technique. Following confirmation of functional PCL based on intraoperative findings, PCL insertion was preserved by creating a bony island.

For the ground KA-TKAs, the distal femoral cut was performed with the assistance of a portable navigation system, followed by the tibial cut using the calipered technique [19]. Before the femoral osteotomies, minimum medial release (osteophyte removal and release of the deep layer of the medial collateral ligament) was performed to maintain medial stability. Femoral osteotomies were performed after correcting for cartilage wear from the distal and posterior femoral condyles equal in thickness (9 mm) to the femoral component; the rotational angle of the femur relative to the posterior condylar axis was set as 0° [12]. Based on the FA value, which was confirmed by preoperative planning and the navigation system, the nTA value was determined by targeting the neutral ground MA, as planned preoperatively. Thus, distal femoral and proximal tibial cuts were performed by referring to preoperative simulation and navigation values.

For the tibia-restricted modified KA-TKAs, the femoral cut was made to be the same as the ground KA-TKA with the assistance of a portable navigation system. Tibial osteotomy was performed at 3° varus relative to the MA and the original posterior slope (up to 10°). Based on a previous report, where the tibial plateau inclination was approximately 3° in asymptomatic volunteers regardless of age, but progressed to approximately 10° with OA progression [20], 3° varus of the tibial cut was applied to avoid severe varus tibial implantation.

### Radiographic measurements

Preoperatively and six months postoperatively, full-length standing coronal radiographs that included the calcaneus (hip-to-calcaneus radiograph) were obtained to evaluate ground MA, as previously described [12, 21]. The patient maintained a unipedal stance on a radiolucent platform and faced a long film cassette. For the lowest point of the calcaneus to be visualized by radiography, the cassettes slid into a position where the lower edge passed through the platform edge. The patient’s patella was placed forward and ankle position was neutral.

Postoperatively, the hip-knee-ankle (HKA) angle and coronal femoral/tibial component alignment (FCA/TCA) during one-leg standing were compared between groups. To determine the intra- and inter-observer reliabilities of the radiographic assessments, the two investigators performed all radiographic assessments twice on 20 randomly selected radiographs. The intra- and inter-observer reliabilities of all radiographic measurements were evaluated using intraclass correlation coefficients (ICCs). The ICCs for intra- and inter-observer reliability were > 0.85 (range, 0.85–0.96) for all measurements. Based on the observed reliability of the results, the measurements obtained by only one of the investigator (N.N.) were used in the analyses.

### Intraoperative measurement of the soft tissue balance

During this study, an offset-type tensor, which enables evaluation of the soft tissue balance throughout the ROM with femoral component placement and patellofemoral joint reduction, was used for the intraoperative measurement of soft tissue balance [22, 23]. This tensor permits intraoperative reproduction of postoperative alignment of the patellofemoral and tibiofemoral joints; the accuracy of measuring this tensor in patients who underwent TKA has been reported [24]. Before the final implantation of the prostheses, the tensor was fixed, and the femoral trial prosthesis and patellofemoral joint were reduced by temporarily suturing the medial parapatellar arthrotomy site. Soft tissue balance, including the joint component gap (mm) and varus/valgus ligament balance (°) with the knee at 0 º, 10 º, 30 º, 60 º, 90 º, and 120 º of flexion were measured with a distraction force of 40 lb. This distraction force was applied several times until the joint centre gap remained constant. This was performed to reduce the error that can result from the creep elongation of the surrounding soft tissues.

### Clinical evaluations

Clinical evaluation was performed for each patient one year postoperatively. The ROM and patient-derived 2011 KSS were assessed, which determined by the patients in the outpatient clinic and includes four categories: symptoms, patient satisfaction, patient expectations, and functional activities. The objective knee indicator score from the 2011 KSS, determined by the surgeon who was blinded to the group assigned, included alignment, instability, and joint motion.

### Statistical analysis

All values were normally distributed and were expressed as mean ± standard deviation (SD). Non-paired *t-*tests were used to compare parameters including radiographic findings, intraoperative soft tissue balance, and clinical outcomes between the groups. Propensity scores were obtained from logistic regression analysis using the following covariates: age, sex, body mass index, preoperative deformity, preoperative ROM, and preoperative clinical scores (objective indicators, patient satisfaction, and functional activity scores). A 1:1 propensity score in nearest neighbour matching without replacement was performed to create matched pairs with the calliper set at 0.2 of a standard deviation of the logit of the propensity score. A standardized difference of < 0.1 was considered adequate. Propensity score matching was conducted using EZR Ver1.36 (Saitama Medical Center, Jichi Medical University, Saitama, Japan), a graphical user interface for R (The R Foundation for Statistical Computing, Vienna, Austria). All other statistical analyses were using a statistical software package (Graph Pad Prism software, Graph Pad, California, USA). Statistical significance was set at* p* < 0.05.

## Results

### Radiographic results

The alignment of the limbs and each component are listed in Table 2. There were no significant differences in the HKA angle and the FCA/ TCA between the groups.

**Table 2:** Postoperative radiological parameters

	Ground KA-TKA	Modified KA-TKA	*p*-value
HKA angle (°)	1.0 ± 1.3 varus	1.6 ± 1.6 varus	0.165
	(1.0 valgus–5.0 varus)	(2.0 valgus–4.0 varus)	
FCA (°)	1.7 ± 1.8 valgus	1.5 ± 1.3 valgus	0.626
	(4.0 valgus–3.0 varus)	(4.0 valgus–2.0 varus)	
TCA (°)	2.8 ± 1.2 varus	3.1 ± 1.0 varus	0.319
	(0–4.5 varus)	(1.0 valgus–4.5 varus)	

Data are presented as the mean ± standard deviation (range). *HKA* hip-knee-ankle, *FCA* femoral component alignment, *TCA* tibial component alignment, *KA-TKA* kinematically aligned total knee arthroplasty

### Intraoperative soft tissue balance

**Fig. 3:**
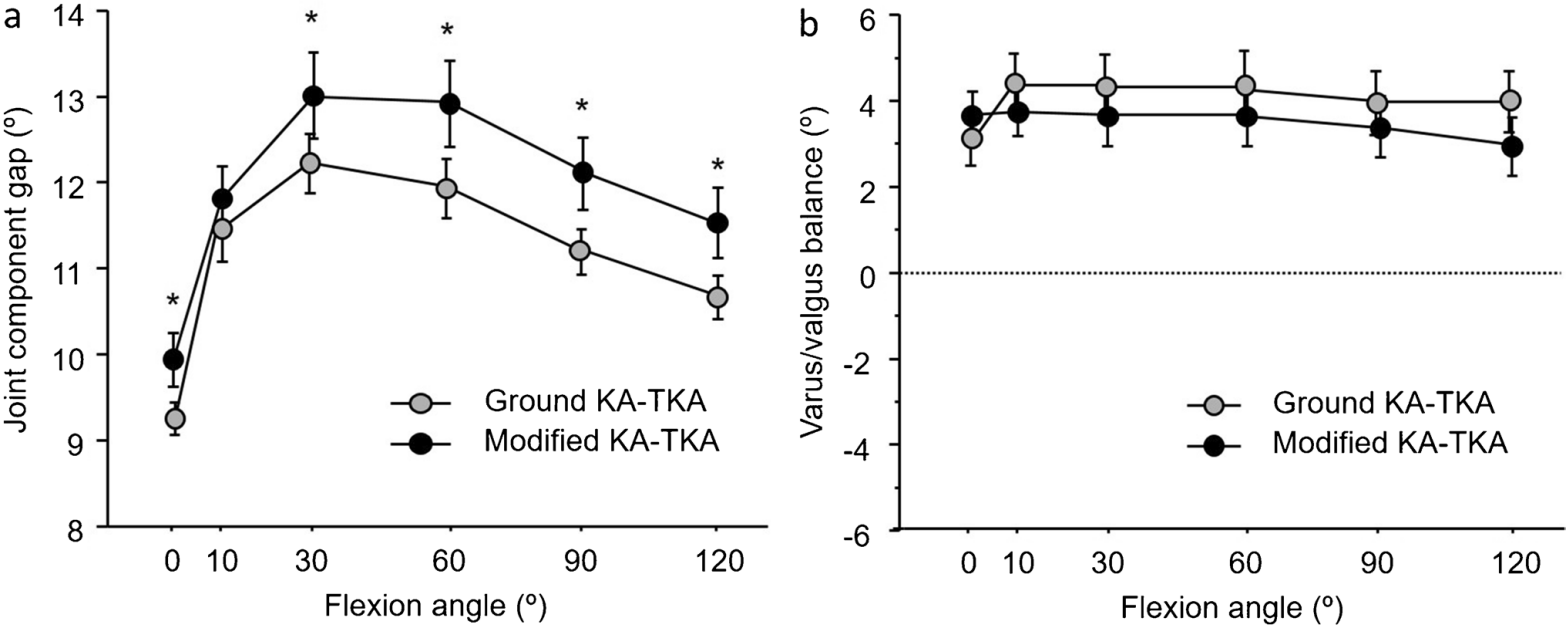
Intraoperative soft tissue balance. **A** Joint component gap. The joint component gaps in participants in the ground KA-TKA group were significantly smaller throughout the range of motion than those in the modified KA-TKA group.*p < 0.05. **B** Varus/valgus ligament balance. The varus/valgus ligament balance in participants exhibited slight lateral laxity throughout the range of motion. The value in both groups showed no significant differences

The mean joint component gaps of the knees are presented in Table 3. The joint component gaps throughout the ROM were significantly smaller in the ground KA-TKA group than in the modified KA-TKA group (Fig. 3a).

**Table 3:** Intraoperative soft tissue balance

	Ground KA-TKA	Modified KA-TKA	*p*-value
Joint component gap (mm)			
0	9.3 ± 0.6	9.9 ± 1.1	0.023
10	11.4 ± 1.0	11.8 ± 1.6	0.340
30	12.2 ± 0.8	13.0 ± 1.7	0.034
60	11.9 ± 0.9	12.9 ± 1.7	0.010
90	11.2 ± 0.7	12.1 ± 1.5	0.009
120	10.7 ± 0.7	11.5 ± 1.4	0.011
Varus/valgus balance (°)			
0	3.1 ± 1.9	3.7 ± 2.2	0.346
10	4.4 ± 2.0	3.8 ± 2.5	0.335
30	4.2 ± 2.1	3.6 ± 2.9	0.401
60	4.3 ± 2.5	3.6 ± 2.9	0.340
90	4.0 ± 2.4	3.3 ± 3.2	0.328
120	4.0 ± 2.5	3.0 ± 2.6	0.187

(Mean ± SD)

*KA-TKA* kinematically aligned total knee arthroplasty

The mean varus/valgus ligament balance (positive value indicates varus balance) of the knee is shown in Table 3. The varus/valgus ligament balance in the participants exhibited slight lateral laxity in both groups, with no significant differences between the groups throughout the ROM (Fig. 3b).

### Clinical results

The average postoperative ROMs and subscales of the 2011 KSS in the ground and modified KA-TKA groups are presented in Table 4. The flexion angles were significantly better in the ground KA-TKA group than in the modified KA-TKA group (*p* = 0.041) (Table 4). All postoperative subscales of the 2011 KSS showed no significant differences between groups (Table 4).

**Table 4:** Postoperative clinical outcomes

	Ground KA-TKA	Modified KA-TKA	*p*-value
ROM			
Extension (°)	− 1.9 ± 2.7	− 1.7 ± 3.0	0.824
	(− 15–0)	(− 10–0)	
Flexion (°)	130.7 ± 6.5	125.7 ± 10.5	0.041
	(110–140)	(85–135)	
2011 KSS			
Objective knee indicators (100)	94.6 ± 4.1	94.9 ± 6.2	0.801
	(86–100)	(79–100)	
Patient satisfaction (40)	28.1 ± 7.6	28.1 ± 7.0	> 0.999
	(14–40)	(12–40)	
Patient expectations (15)	11.5 ± 2.3	11.8 ± 3.2	0.744
	(6–15)	(5–15)	
Functional activities (100)	78.8 ± 11.6	77.8 ± 18.1	0.797
	(63–100)	(66–96)	

(mean ± SD (range))

*ROM* range of motion, *KSS* Knee Society Score, *KA-TKA* kinematically aligned total knee arthroplasty

## Discussion

The most important finding of this study was that for patients with varus OA, ground KA-TKA achieved smaller joint component gaps throughout the ROM and better flexion angle than tibia-restricted modified KA-TKA, as partially hypothesized. To the best of our knowledge, this study is the first to report the intraoperative soft tissue balance and early clinical outcomes of this new KA-TKA technique that targets neutral ground MA.

In KA-TKA, the physiological joint line and kinematics are ideal for reproducing a pre-arthritic knee. In contrast, pre-arthritic knees and young healthy knees demonstrate slight varus alignment on average, constitutional varus, in conventional MA assessment [8, 25]. This raises the question of why healthy young knees are not in neutral MA. Haraguchi et al. suggested that true MA, previously called ground MA [6, 7], should be assessed from the hip centre to the lowest point of the calcaneus, not the ankle centre [26]. Tanaka et al. reported 34 healthy individuals (mean age, 26.4 years) with ground MA ratios of 51.4% and 50.4% and conventional MA ratios of 46.3% and 46.1% in single-leg and double-leg standing positions, respectively. This indicated that ground MA should be considered for the assessment or planning of knee osteotomy and reconstruction surgeries [8]. Kikuchi et al. reported that in 21 patients with varus OA, ground MA was more closely correlated with knee adduction angular impulse than conventional MA [9]. Thus, our philosophy of targeting the neutral ground MA in KA-TKA is reasonable.

In this study, the ground KA-TKA group exhibited more stable joint component gaps throughout the ROM than the modified KA-TKA group. In both procedures, the femoral cut was performed in the same way according to the calipered method. Therefore, difference may be due to the difference in the tibial cut procedures; the tibia was cut individually to the femoral cut in the ground KA-TKA, whereas the systemic tibial cut (3° in varus) was performed in the modified KA-TKA. This difference in the tibial cut may lead to a smaller distribution (standard deviation) in ground KA-TKA, as shown in Table 3, resulting in smaller and more stable joint component gaps. This stable joint component gap in ground KA-TKA may result in a higher flexion angle. It has been previously reported that an excessively loose flexion gap relative to the extension gap should be avoided to obtain a high flexion angle in TKA [27]. Our current findings indicate that joint component gaps in ground KA-TKA might be the appropriate level of tension. In ground KA-TKA, appropriate joint component gaps and slight lateral laxity may lead to a high flexion angle.

This study had several limitations. First, the assessments were performed in a small patient population, and those with severe varus, valgus deformities, and ankle/foot deformities were excluded. The large number of patients with valgus and severe deformities may have influenced the results and should be examined in the future. In addition, the clinical outcomes were assessed only one year after the surgeries. This may not have resulted in true clinical relevance, and longer-term follow-up should be assessed.

In conclusion, the ground KA-TKA technique for patients with mild-to-moderate varus OA resulted in more stable joint component gaps throughout the ROM, and a better flexion angle when compared with tibia-restricted modified KA-TKA. This new KA-TKA procedure, as a personalized alignment technique, may provide physiological alignment more comparable to that of the native knee than that of other TKA alignment techniques.

## References

[1] Hirschmann MT, Karlsson J, Becker R (2018). Hot topic: alignment in total knee arthroplasty-systematic versus more individualised alignment strategies. Knee Surg Sports Traumatol Arthrosc.

[2] Howell SM, Howell SJ, Kuznik KT, Cohen J, Hull ML (2013). Does a kinematically aligned total knee arthroplasty restore function without failure regardless of alignment category?. Clin Orthop Relat Res.

[3] Hutt JR, LeBlanc MA, Masse V, Lavigne M, Vendittoli PA (2016). Kinematic TKA using navigation: Surgical technique and initial results. Orthop Traumatol Surg Res.

[4] Courtney PM, Lee GC (2017). Early outcomes of kinematic alignment in primary total knee arthroplasty: a meta-analysis of the literature. J Arthroplasty.

[5] Hiyama S, Takahashi T, Takeshita K (2022). Kinematically aligned total knee arthroplasty did not show superior patient-reported outcome measures: an updated meta-analysis of randomized controlled trials with at least 2-year follow-up. J Knee Surg.

[6] Mullaji A, Shetty GM (2011). Persistent hindfoot valgus causes lateral deviation of weightbearing axis after total knee arthroplasty. Clin Orthop Relat Res.

[7] Desai SS, Shetty GM, Song HR, Lee SH, Kim TY, Hur CY (2007). Effect of foot deformity on conventional mechanical axis deviation and ground mechanical axis deviation during single leg stance and two leg stance in genu varum. Knee.

[8] Tanaka T, Takayama K, Hashimoto S, Kanzaki N, Hayashi S, Kuroda R, Matsumoto T (2017). Radiographic analysis of the lower limbs using the hip-calcaneus line in healthy individuals and in patients with varus knee osteoarthritis. Knee.

[9] Kikuchi N, Kanamori A, Kadone H, Okuno K, Hyodo K, Yamazaki M (2022). Radiographic analysis using the hip-to-calcaneus line and its association with lower limb joint kinetics in varus knee osteoarthritis. Knee.

[10] Kim JG, Suh DH, Choi GW, Koo BM, Kim SG (2021). Change in the weight-bearing line ratio of the ankle joint and ankle joint line orientation after knee arthroplasty and high tibial osteotomy in patients with genu varum deformity. Int Orthop.

[11] Naylor BH, Seidman D, Scuderi GR (2021). Bridging the gap: the influence of foot and ankle pathomechanics in total knee arthroplasty. J Am Acad Orthop Surg.

[12] Matsumoto T, Takayama K, Ishida K, Hayashi S, Hashimoto S, Kuroda R (2017). Radiological and clinical comparison of kinematically versus mechanically aligned total knee arthroplasty. Bone Joint J.

[13] Kamenaga T, Nakano N, Takayama K, Tsubosaka M, Takashima Y, Kikuchi K, Fujita M, Kuroda Y, Hashimoto S, Hayashi S, Niikura T, Kuroda R, Matsumoto T (2021). Comparison of plantar pressure distribution during walking and lower limb alignment between modified kinematically and mechanically aligned total knee arthroplasty. J Biomech.

[14] Matsumoto T, Takayama K, Ishida K, Kuroda Y, Tsubosaka M, Muratsu H, Hayashi S, Hashimoto S, Matsushita T, Niikura T, Kuroda R (2020). Intraoperative Soft tissue balance/kinematics and clinical evaluation of modified kinematically versus mechanically aligned total knee arthroplasty. J Knee Surg.

[15] Matsumoto T, Nakano N, Ishida K, Maeda T, Tachibana S, Kuroda Y, Hayashi S, Matsushita T, Kuroda R (2023) Targeting the neutral hip-to-calcaneus axis in kinematically aligned total knee arthroplasty is feasible with fewer alignment outliers for varus osteoarthritic patients. Knee Surg Sports Traumatol Arthrosc. 10.1007/s00167-023-07306-1 10.1007/s00167-023-07306-1 PMC10435616 36918435

[16] Cho WS, Cho HS, Byun SE (2017). Changes in hindfoot alignment after total knee arthroplasty in knee osteoarthritic patients with varus deformity. Knee Surg Sports Traumatol Arthrosc.

[17] Norton AA, Callaghan JJ, Amendola A, Phisitkul P, Wongsak S, Liu SS, Fruehling-Wall C (2015). Correlation of knee and hindfoot deformities in advanced knee OA: compensatory hindfoot alignment and where it occurs. Clin Orthop Relat Res.

[18] Scuderi GR, Bourne RB, Noble PC, Benjamin JB, Lonner JH, Scott WN (2012). The new knee society knee scoring system. Clin Orthop Relat Res.

[19] Howell SM, Papadopoulos S, Kuznik KT, Hull ML (2013). Accurate alignment and high function after kinematically aligned TKA performed with generic instruments. Knee Surg Sports Traumatol Arthrosc.

[20] Matsumoto T, Hashimura M, Takayama K, Ishida K, Kawakami Y, Matsuzaki T, Nakano N, Matsushita T, Kuroda R, Kurosaka M (2015). A radiographic analysis of alignment of the lower extremities–initiation and progression of varus-type knee osteoarthritis. Osteoarthr Cartil.

[21] Reilingh ML, Beimers L, Tuijthof GJ, Stufkens SA, Maas M, van Dijk CN (2010). Measuring hindfoot alignment radiographically: the long axial view is more reliable than the hindfoot alignment view. Skeletal Radiol.

[22] Matsumoto T, Muratsu H, Tsumura N, Mizuno K, Kuroda R, Yoshiya S, Kurosaka M (2006). Joint gap kinematics in posterior-stabilized total knee arthroplasty measured by a new tensor with the navigation system. J Biomech Eng.

[23] Muratsu H, Matsumoto T, Kubo S, Maruo A, Miya H, Kurosaka M, Kuroda R (2010). Femoral component placement changes soft tissue balance in posterior-stabilized total knee arthroplasty. Clin Biomech (Bristol, Avon).

[24] Matsumoto T, Kuroda R, Kubo S, Muratsu H, Mizuno K, Kurosaka M (2009). The intra-operative joint gap in cruciate-retaining compared with posterior-stabilised total knee replacement. J Bone Joint Surg Br.

[25] Bellemans J, Colyn W, Vandenneucker H, Victor J (2012). The Chitranjan Ranawat award: is neutral mechanical alignment normal for all patients? The concept of constitutional varus. Clin Orthop Relat Res.

[26] Haraguchi N, Ota K, Tsunoda N, Seike K, Kanetake Y, Tsutaya A (2015). Weight-bearing-line analysis in supramalleolar osteotomy for varus-type osteoarthritis of the ankle. J Bone Joint Surg Am.

[27] Matsumoto T, Mizuno K, Muratsu H, Tsumura N, Fukase N, Kubo S, Yoshiya S, Kurosaka M, Kuroda R (2007). Influence of intra-operative joint gap on post-operative flexion angle in osteoarthritis patients undergoing posterior-stabilized total knee arthroplasty. Knee Surg Sports Traumatol Arthrosc.

